# Changes in maintenance immunosuppression after pediatric kidney transplantation—a report from the Nordic pediatric kidney transplantation registry

**DOI:** 10.1007/s00467-025-07030-7

**Published:** 2025-11-13

**Authors:** Henna Kaijansinkko, Juuso Tainio, Anna Bjerre, Ann Christin Gjerstad, Ilse D. S. Weinreich, Hannu Jalanko, Lars Wennberg, Susanne Westphal Ladfors, Helle Charlotte Thiesson, Zivile Bekassy, Søren Schwartz Sørensen, Timo Jahnukainen

**Affiliations:** 1 Department of Pediatrics, Tampere University Hospital, Tampere, Finland; 2 Department of Pediatric Nephrology and Transplantation, New Children’s Hospital, University of Helsinki and Helsinki University Hospital, Helsinki, Finland; 3 Division of Pediatric and Adolescent Medicine, Oslo University Hospital, Oslo, Norway; 4 Scandiatransplant Office, Aarhus University Hospital, Aarhus, Denmark; 5 Department of Clinical Science, Intervention and Technology, Department of Transplantation, Karolinska University Hospital, Stockholm, Sweden; 6 Department of Pediatrics, The Queen Silvia Children’s Hospital, Sahlgrenska University Hospital, Gothenburg, Sweden; 7 Department of Nephrology, Odense University Hospital, Odense, Denmark; 8 Department of Pediatric Nephrology, Skane University Hospital, Lund, Sweden; 9 Department of Nephrology, Rigshospitalet, Copenhagen University Hospital, Copenhagen, Denmark; 10 Department of Clinical Medicine, University of Copenhagen, Copenhagen, Denmark

**Keywords:** Kidney transplantation, Immunosuppression, Pediatric, Cyclosporine A, Tacrolimus, Mycophenolate mofetil

## Abstract

**Background:**

Few studies are available on changes in maintenance immunosuppression after pediatric kidney transplantation (KT). This is a retrospective registry analysis of the long-term medication modifications in the Nordic countries.

**Methods:**

All pediatric KT recipients transplanted between the years 2005 and 2016 were identified from the Scandiatransplant registry. Of the 482 patients, 345 met the inclusion criteria: age below 16 years at KT and at least 2 years post-transplant follow-up.

**Results:**

A change in maintenance immunosuppression occurred in 160 patients (46.4%) at 2.0 (interquartile range 1.0–3.0) years median time from KT. The most common change (35.8%) was switching cyclosporine A (CsA) to tacrolimus (Tac). Initial CsA treatment was modified significantly more often compared to Tac (72.0% vs. 6.0%; *p* < 0.001). Modifications of mycophenolate mofetil (MMF) were observed more often in recipients aged < 2 (75.0%) and 2–5 (55.6%) years compared with 5–16 years (13.2%; *p* < 0.001); particularly, MMF discontinuation was common (< 2 years 45.8% and 2–5 years 38.9%). Otherwise, initial immunosuppression remained mainly unchanged. The main reasons for changing CsA to Tac were cosmetic side effects (26.2%), rejections (26.2%), and declining graft function (23.0%). In case of rejection or declining graft function**,** CsA-to-Tac conversion slowed the decrease in measured glomerular filtration rate. MMF modifications did not affect graft survival from 2 to 7.5 years post-transplant.

**Conclusions:**

Maintenance immunosuppression is modified in almost half of pediatric KT recipients. Particularly, CsA conversion to Tac and young recipients’ MMF modifications are common.

**Graphical abstract:**

**Figure:**
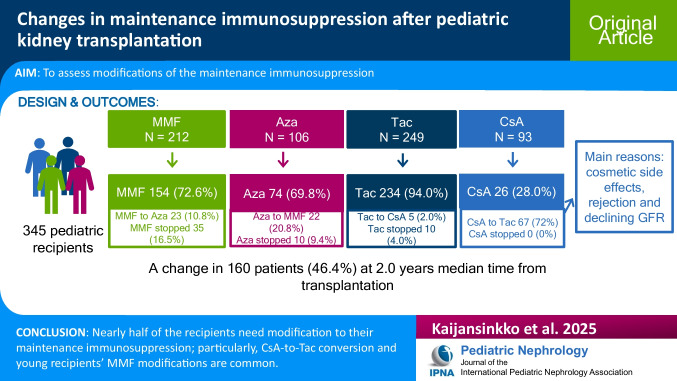
A higher resolution version of the Graphical abstract is available as Supplementary information

**Supplementary Information:**

The online version contains supplementary material available at 10.1007/s00467-025-07030-7.

## Introduction

Despite the development of new immunosuppressive drugs, such as mammalian target of rapamycin (mTOR) inhibitors and inhibitors of co-stimulation, the conventional combination of calcineurin inhibitors (CNI) and antimetabolites, with or without glucocorticoids, has remained the backbone of immunosuppression (IS) after kidney transplantation (KT). During the past decades, tacrolimus (Tac) and mycophenolate mofetil (MMF) have largely replaced cyclosporine A (CsA) and azathioprine (Aza) as the main IS drugs in pediatric KT recipients [1–3]. Several adult and pediatric studies have confirmed better efficacy of Tac over CsA in terms of graft survival and rejection rates [4–6]. Using the same criteria, Tac and MMF form the best IS combination in adults [7]. Using Tac + MMF IS, early or late steroid withdrawal does not affect graft or patient survival, even though the number of rejections increases in adults [8–10]. In pediatric recipients, even the rejection rate remains comparable, according to large registry data [11].

While KT outcomes have improved, we still face problems with the IS adverse effects [12]. Adjustment of the IS level remains a double-edged sword. With under-immunosuppression, the patient is exposed to cell- and antibody-mediated rejection, while over-immunosuppression carries risks of viral and bacterial infections and malignancies [13, 14]. Post-transplant lymphoproliferative disorder (PTLD) prevalence is approximately 2–4% among pediatric KT recipients [1, 15]. Recipient’s young age and EBV-seronegativity are known risk factors for PTLD [16]. In addition, cosmetic side effects, such as hypertrichosis and gingival hyperplasia, are common with CsA, while cytopenias and gastrointestinal symptoms might cause problems with Tac and MMF [12, 17].

Knowledge of maintenance IS modifications in pediatric KT recipients is scarce. Therefore, we aimed to evaluate changes in the maintenance IS regimen from the Scandiatransplant registry during long-term follow-up; we also aimed to assess reasons for the drug modifications and their possible influence on graft function.

## Patients and methods

### Data source and definition of variables

The Scandiatransplant pediatric kidney transplantation sub-registry (Nordic Pediatric Renal Transplant Study Group, NPRTSG) was founded in 1994 [18]. Each transplant center within the Scandiatransplant region (Denmark, Estonia (since 2017), Finland, Iceland, Norway, and Sweden) has an obligation to report baseline and follow-up data to the registry. The data contain information about every transplantation performed on patients below the age of 16 years (age, diagnosis, donor type, initial IS, human leukocyte antigen (HLA), and blood group (ABO) compatibility) and annual follow-up data consisting of maintenance IS, rejections, graft survival, plasma creatinine level, glomerular filtration rate (GFR), and malignancies.

For the present study, data on KTs performed between January 1, 2005, and December 31, 2016, were collected retrospectively from the Scandiatransplant registry. IS data were collected, if available, until the end of study on November 8, 2019, transition to adult nephrology or death. Patients with a minimum of 2-year follow-up were included in the data analyses. A total of 482 pediatric patients were transplanted in the Nordic countries during the study era; the follow-up IS data were available for 345 (71.6%) of them. IS at discharge after KT was defined as the initial IS. While discontinuation and switch of drugs were counted as changes, dose reductions were not collected or analyzed. Modifications in steroid treatment were not counted as IS changes and were analyzed separately. Early steroid withdrawal was defined as not having steroids at discharge and late steroid withdrawal as steroid discontinuation thereafter. From the registry, only concurrent rejection or malignancy could be identified as a reason for drug change.

However, the data were complemented regarding CsA-to-Tac conversions. Most shifts from CsA to Tac (*n* = 61; 91%) were done in one center (Helsinki, Finland); for this subgroup of patients, reasons for CNI conversion and measured glomerular filtration rate (mGFR) before and after the conversion were requested. Data containing reasons for the drug changes were received from 61 recipients. The GFR values, which were measured either with Cr-EDTA or technetium-99 m-EDTA clearance at 3, 6, 12, 18, and 24 months after KT and once per year thereafter until the end of follow-up (up to 15 years), were obtained from 49 recipients. The mean annual rate of change before and after CsA-to-Tac conversion was calculated for mGFR (mL/min/1.73 m^2^/year). Rejections were histologically confirmed. However, the definition according to the Banff classification was available only in a subset of patients [19]. All reported and treated rejections were included in the analysis. Drug modification groups did not exist at KT but formed in the following years. Thus, early graft losses could not enroll in these groups. For this reason, we analyzed graft survival from two years on.

### Statistics

Data on initial IS and induction therapy were collected and analyzed drug by drug and as IS combinations. HLA-A, B, and DRB1 mismatches were analyzed as sum mismatches. Their impact on initial CNI choice was studied using Fisher’s exact test in two groups: < 4 mismatches compared to ≥ 4 mismatches. The Kruskal-Wallis test and Mann-Whitney tests with Bonferroni correction in pairwise comparisons were performed to study initial IS combinations’ impact on the number of rejections. Binary logistic regression was performed to study the association between initial CNI and PTLD and the association between age groups and PTLD. The number of PTLD cases did not allow adjustment for confounders. Steroid withdrawal’s effect on the number of rejections was studied using the Kruskal-Wallis test, and the test was repeated by limiting the analysis to patients with Tac + MMF as initial IS. Data on IS changes were analyzed based on initial IS and reported maintenance IS. Time from KT to the first IS modification was assessed. Initial CNI and antimetabolite permanence (change overall vs. no change) and specific drug permanence (change overall vs. no change) in age groups and according to transplant era were studied using Fisher’s exact test. In the subgroup of patients with CsA-to-Tac conversion, the rate of change in mGFR per year before and after the drug switch was calculated and compared using the Wilcoxon signed-rank test. The incidence rate of rejection in person-years of follow-up was calculated for MMF-continued and MMF-modified groups, as well as before and after the MMF modification in the MMF-modified group. The incidence rates were compared using a Poisson regression model adjusting for age groups; for repeated measures, comparison was performed using a generalized estimating equation. Death-censored graft survival was analyzed between 2 and 7.5 years for the MMF-modified and the MMF-continued groups and compared using a log-rank test. *p*-values < 0.05 were regarded as statistically significant. All data were analyzed using SPSS 28.0 (SPSS Inc., Chicago, IL, USA).

### Ethics

The registry data collection and extraction were done by Scandiatransplant. Data analyses were performed at the New Children’s Hospital, Helsinki University Hospital. The Research Ethics Committee of Helsinki and Uusimaa Hospital District approved the study (HUS 939/2018). The study was a retrospective registry-based analysis, and according to current regulations, no informed written consent from study subjects or their caregivers was required.

## Results

### Patient characteristics

The mean age at KT was 7.9 (standard deviation, SD 5.1) years, and the median follow-up time after KT was 5.0 (interquartile range, IQR 3.0–8.0) years. Primary diagnoses leading to KT are presented in Table 1. Congenital anomalies of the kidney and urinary tract (CAKUT), including obstructive uropathy, reflux nephropathy, kidney hypoplasia, and dysplasia, were the leading causes for KT, accounting for 30.5% of the cases.

**Table 1:** Demographics of the 345 pediatric kidney transplant recipients included in the study

Median follow-up time, years (IQR; range)	5.0 (3.0–8.0; 2.0–14.0)
Transplant era, *n* (%)	
2005–2010	195 (56.5)
2011–2016	150 (43.5)
Mean age at transplantation, years (SD)	7.9 (5.1)
Primary diagnosis, *n* (%)	
CAKUT	90 (30.5)
CNS	39 (13.2)
Cystic diseases	39 (13.2)
Glomerulonephritis	13 (4.4)
FSGS	12 (4.1)
HUS/aHUS	6 (2.0)
Vascular glomerulopathies	3 (1.0)
Miscellaneous	93 (31.5)
Unknown	50
Sex, *n* (%)	
Female	150 (43.5)
Male	195 (56.5)
Country (centers), *n* (%)	
Sweden (Stockholm, Gothenburg, Skåne, Uppsala)	111 (32.2)
Finland (Helsinki)	100 (29.0)
Denmark (Copenhagen, Odense, Aarhus)	83 (24.1)
Norway (Oslo)	51 (14.8)
Donor type, *n* (%)	
LD	201 (58.3)
DD	144 (41.7)
HLA mismatches, *n* (%)	
0–2	126 (39.5)
3–4	164 (51.4)
5–6	29 (9.1)
Unknown	26
HLA immunization, *n* (%)	
Not immunized (PRA = 0%)	230 (80.7)
Lowly immunized (PRA 1–9%)	16 (5.6)
Immunized (PRA 10–79%)	22 (7.7)
Highly immunized (PRA ≥ 80%)	3 (1.1)
Previously immunized	14 (4.9)
Unknown	60
ABOi, *n* (%)	17 (4.9)
Primary transplantation, *n* (%)	325 (94.2)

*IQR* interquartile range, *SD* standard deviation, *CAKUT* congenital anomalies of the kidney and urinary tract (including kidney hypo/dysplasia, obstructive uropathy and reflux nephropathy), *CNS* congenital nephrotic syndrome, *FSGS* focal segmental glomerulosclerosis, *HUS* hemolytic uremic syndrome, *aHUS* atypical hemolytic uremic syndrome, *LD* living donor, *DD* deceased donor, *HLA* human leucocyte antigen, *PRA* panel reactive antibody, *ABOi* ABO incompatible

### Initial immunosuppression

Basiliximab or antithymocyte globulin (ATG) were given to 50.7% and 9.9% of the recipients as induction therapy, respectively (Table 2). Most patients had Tac and MMF (55.4%) as an initial IS combination. However, for patients under 2 years of age, the combination of CsA and Aza (*n* = 35; 48.6%) was the most common choice. ABO incompatible transplantations were treated with a Tac, MMF, and steroid combination. Patients who had four or more HLA-A, B, and DRB1 mismatches were more likely to have Tac as the initial IS (≥ 4 mismatches 84.4% vs. < 4 mismatches 69.4%; *p* = 0.014; Supplementary Table 1 shows sum mismatches and initial IS). Immunosuppressive protocols varied somewhat between centers. Tac and MMF were utilized in all countries. However, 91.4% and 77.4% of CsA and Aza initiations, respectively, were conducted at a single center in Helsinki, Finland. Early steroid withdrawals were performed at one center in Odense, Denmark.

**Table 2:** Induction agent, initial combination of maintenance immunosuppressive drugs, and the use of steroids at discharge

Initial IS	*n* (%)
Biologic induction agent	
Basiliximab	154 (50.7)
None	120 (39.5)
ATG	30 (9.9)
Unknown	41
IS combination	
Tac + MMF	191 (55.4)
CsA + Aza	71 (20.6)
Tac + Aza	35 (10.1)
CsA + MMF	18 (5.2)
Other	30 (8.7) ^a^
Use of steroids	
Steroid	302 (88.3)
No steroid	40 (11.7)
Unknown	3

*IS* immunosuppression, *ATG* anti-thymocyte globulin, *Tac* tacrolimus, *MMF* mycophenolate mofetil, *CsA* cyclosporine A, *Aza* azathioprine

^a^Including mainly patients with CNI ± steroids but without antimetabolite. None of the patients had mammalian target of rapamycin as initial immunosuppression

In our cohort, CsA and Aza as an initial IS resulted in more rejections compared to Tac and MMF (*p* = 0.013; Supplementary Table 2). PTLD cases were observed less in the oldest patient group compared to the youngest group (5–16 years vs. < 2 years OR = 0.22; 95% CI 0.05–0.93; *p* = 0.040; Supplementary Table 3).

### Steroid withdrawal

Early steroid withdrawal before discharge was performed in 40 (11.7%) patients. For nine of those patients, steroid treatment was reinstated later during follow-up, including two with reported rejection. Late steroid withdrawal was observed in 27 (7.9%) patients, while the majority (275, 80.4%) were maintained on steroid treatment throughout the follow-up. Initial IS was Tac + MMF in 95.0% and 74.1% of early and late steroid withdrawal patients, respectively. Patients in the early steroid withdrawal group received basiliximab or ATG as induction therapy, while 33.3% of patients with late steroid withdrawal received no induction. The number of rejections did not differ significantly between patients with early steroid withdrawal, late steroid withdrawal, or long-term steroid treatment (*p* = 0.246); a similar result was obtained when analysis was limited to patients with Tac + MMF as the initial IS (*p* = 0.359; Supplementary Table 4).

### Changes in maintenance immunosuppression

**Fig. 1:**
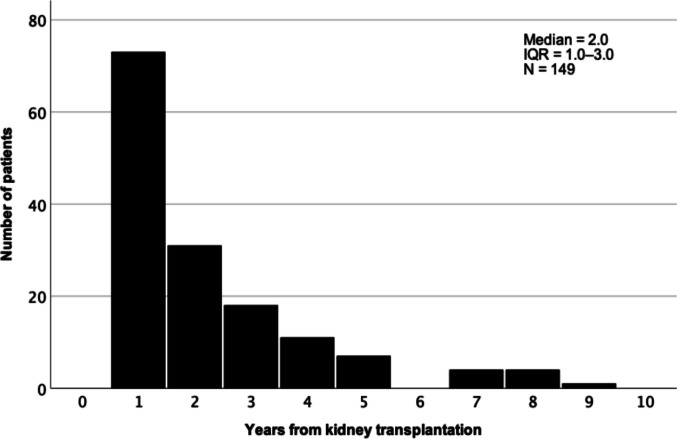
Time from kidney transplantation to the first immunosuppression modification. IQR, interquartile range. No data on time of immunosuppression change in 11 patients

Maintenance IS was modified in 160 patients (46.4%). At 2 and 5 years post-transplant, only 241 of 345 (69.9%) and 93 of 201 (46.3%) patients still used their initial IS, respectively. The most common IS change was a switch from CsA to Tac, accounting for 35.8% of all changes. In all, our data contained 22 different changes or combinations of changes. Multiple changes in maintenance IS were observed in 27 recipients. Over half of the first IS modifications occurred before 2 years post-transplant (median 2.0; IQR 1.0–3.0 years; Fig. 1). The time from KT to modification was similar in all prevalent modifications (CsA to Tac, Aza to MMF, MMF to Aza, and antimetabolite discontinuations): modifications predominantly occurred in the early years following KT, with relatively few taking place in later years. Immunosuppression changes did not differ between transplant eras (Supplementary Table 5). PTLD was identified as a reason for the IS change in 9 (5.7%) recipients.

CsA was converted to Tac in 66.7%, 78.6%, and 75.7% of recipients aged < 2 years, 2–5 years, and 5–16 years, respectively (Table 3). Modifications were observed significantly more often in CsA treatment compared to Tac treatment (*p* = 0.001). Concurrent rejection was identified in 17.0% of all IS-modified patients and in 23.9% of CsA-to-Tac-converted patients, according to registry data (Supplementary Table 6).

**Table 3:** Changes in initial maintenance immunosuppression; the data are presented in all kidney transplant recipients and in subgroups according to the patient age at the time of transplantation

Immunosuppressive drug	** < **2 years *n* = 72	2–5 years *n* = 58	5–16 years *n* = 215	*p*-value	Total *n* = 345	*p*-value
Calcineurin inhibitors						*p* < 0.001^a^
Cyclosporine A, *n* (%)				*p* = 0.603 ^b^		
No change	14 (33.3)	3 (21.4)	9 (24.3)		26 (28.0)	
CsA → Tac	28 (66.7)	11 (78.6)	28 (75.7)		67 (72.0)	
CNI stopped	0 (0)	0 (0)	0 (0)		0 (0)	
Tacrolimus, *n* (%)				*p* = 0.114 ^b^		
No change	25 (86.2)	41 (93.2)	168 (95.5)		234 (94.0)	
Tac → CsA	2 (6.9)	1 (2.3)	2 (1.1)		5 (2.0)	
CNI stopped	2 (6.9)	2 (4.5)	6 (3.4)		10 (4.0) ^c^	
No CNI, *n* (%)	1 (1.4)	0 (0)	2 (0.9)		3 (0.9)	
Antimetabolites						*p* = 0.600 ^d^
MMF, *n* (%)				*p* < 0.001^e^		
No change	6 (25.0)	16 (44.4)	132 (86.8)		154 (72.6)	
MMF → Aza	7 (29.2)	6 (16.7)	10 (6.6)		23 (10.8)	
Antimetabolite stopped	11 (45.8)	14 (38.9)	10 (6.6)		35 (16.5) ^c^	
Aza, *n* (%)				*p* = 1.000 ^b^		
No change	31 (68.9)	10 (71.4)	33 (70.2)		74 (69.8)	
Aza → MMF	7 (15.6)	3 (21.4)	12 (25.5)		22 (20.8)	
Antimetabolite stopped	7 (15.6)	1 (7.1)	2 (4.3)		10 (9.4)	
No antimetabolite, *n* (%)	3 (4.2)	8 (13.8)	16 (7.4)		27 (7.8)	

*CsA* cyclosporine A, *CNI* calcineurin inhibitor, *Tac* tacrolimus, *MMF* mycophenolate mofetil, *Aza* azathioprine

^a^ Fisher’s exact test. Statistically significant difference in changes (no change vs. change) between CsA and Tac

^b^ Fisher’s exact test. No statistically significant difference in changes (no change vs. change) between age groups

^c^ Including 4 patients who were converted to mammalian target of rapamycin

^d^ Fisher’s exact test. No statistically significant difference in changes (no change vs. change) between MMF and Aza

^e^ Fisher’s exact test. Statistically significant difference in changes (no change vs. change) in  5–16 years age group compared to younger age groups

Change in analyses included drug switch and discontinuation

MMF treatment was modified significantly more often in recipients aged < 2 years (75.0%) and 2–5 years (55.6%) compared to 5–16 years (13.2%; p = 0.001; Table 3). Discontinuation was common in recipients aged < 2 years (45.8%) and 2–5 years (38.9%). Of all patients, 72.6% starting with MMF and 69.8% starting with Aza continued with the initial antimetabolite throughout the follow-up.

### Subgroup analysis of reasons for CsA-to-Tac conversion and subsequent change in mGFR

The subgroup analysis of reasons for switching from CsA to Tac included 61 patients and covered 91% of all CsA-to-Tac conversions. The median time to conversion after KT was 1.2 years (range 0.2–7.2). The leading causes were cosmetic side effects (26.2%), rejections (26.2%), and rising creatinine level combined with interstitial fibrosis and tubular atrophy (IFTA) in allograft biopsy (23.0%) (Table 4).

**Table 4:** Reasons for switching cyclosporine A to tacrolimus in the Finnish subgroup of pediatric kidney transplant recipients

Reason for drug change	Number of patients (%) *N* = 61
Cosmetic reason	16 (26.2)
Rejection	16 (26.2)
Decreasing GFR and IFTA	14 (23.0)
Unknown	6 (9.8)
Re-nephrosis	3 (4.9)
Viremia	1 (1.6)
Unstable drug level	1 (1.6)
TMA	1 (1.6)
De novo DSA	1 (1.6)
GI symptoms	1 (1.6)
Error in registry	1 (1.6)

*GFR* glomerular filtration rate, *IFTA* interstitial fibrosis and tubular atrophy,  *TMA* thrombotic microangiopathy, *DSA* donor specific antibody, *GI* gastrointestinal

Measured GFR decreased significantly less after conversion from CsA to Tac if the conversion was performed due to rejection or decline in mGFR (*n* = 20; median 5.4 mL/min/1.73 m^2^/year (IQR 1.0–21.9) vs. 0.6 mL/min/1.73 m^2^/year (IQR −3.1–3.0); *p* = 0.006) but remained stable if CsA was converted due to other causes (*n* = 14; median 0.7 mL/min/1.73 m^2^/year (IQR −2.2–13.5) vs. median 1.9 mL/min/1.73 m^2^/year (IQR −1.1–3.4); *p* = 0.98).

### Outcomes after MMF modification

**Fig. 2:**
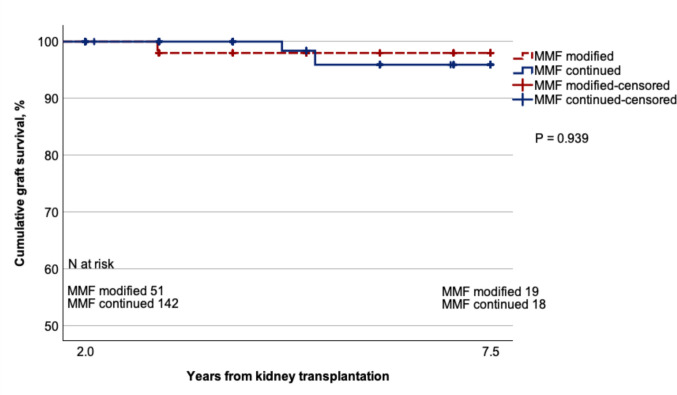
Graft survival probability in the MMF-continued and MMF-modified groups between 2 and 7.5 years after kidney transplantation. MMF, mycophenolate mofetil; N, number

A trend toward a lower age-adjusted risk of rejection per person-year of follow-up was observed in the MMF-modified group (mean 0.02 rejections/person-year; 95% CI 0.01–0.04) compared to the MMF-continued group (mean 0.04 rejections/person-year; 95% CI 0.02–0.08), suggesting potential selection bias (RR 0.48; 95% CI 0.21–1.09; *p* = 0.079). The incidence rate of rejection did not increase after MMF modifications (after mean 0.009 rejections/person-year (95% CI 0.001–0.145) vs. before mean 0.040 rejections/person-year (95% CI 0.017–0.095); RR 0.23; 95% CI 0.016–3.251; *p* = 0.275). All three rejections recorded after the MMF modification occurred in a single patient. From 2 to 7.5 years after KT, graft survival was comparable between the MMF-continued and MMF-modified groups (*p* = 0.939; Fig. 2). At 7.5 years follow-up (started from 2 years on), graft survival was 96% and 98% in the MMF-continued and MMF-modified groups, respectively.

## Discussion

Selection of maintenance IS after solid organ transplantation is traditionally based on each transplant center’s treatment protocol. In recent years, individualized IS has become more common instead of fixed protocols. In the present study, a change in maintenance IS was noticed in 46.4% of the patients. The most frequent modification was a switch from CsA to Tac, and the most common reasons for this CNI conversion were cosmetic side effects, rejections, and decreasing GFR combined with IFTA. Another major need for modifications was observed in young recipients’ MMF treatment. If CsA was switched to Tac due to rejection or decline in mGFR, the decrease in mGFR slowed down after the switch. MMF modifications did not affect the graft survival.

Our data show a decreased number of rejections in children with Tac + MMF combination compared to those with CsA + Aza medication and no difference in steroid withdrawal. On the other hand, our data also exhibit a higher prevalence of PTLD in young recipients, but no difference according to the initial CNI. Thus, our data before IS modifications are comparable with previous studies [4, 6, 11, 20–22].

Patients who have CsA as an initial CNI are mostly converted to Tac relatively soon after KT, whereas Tac is rather stable as an initial CNI. Though the CsA conversion rate is known to be high in pediatric recipients (41.2%) [12], it was even higher (72.0%) in our study. Cosmetic side effects, including hypertrichosis and gingival hyperplasia, are the major reasons for CsA discontinuation. Young children often do not experience distress because of these side effects, but for teenagers, their impact can be drastic. In our study, cosmetic side effects were the reason for switching from CsA to Tac in 26.2% of cases, which is similar to an earlier report by Lancia et al. [12].

The present study suggests that the conversion from CsA to Tac reduces the decline in mGFR when the change is made due to decreasing GFR or IFTA. Our finding regarding mGFR aligns with a previous adult study where GFR improved 12 months after the conversion in chronic allograft nephropathy [23]. Besides a CsA-to-Tac conversion, optimizing treatment with MMF would be another reasonable option in a situation of declining GFR, but that was not analyzed in the present study [13, 24].

MMF modifications are common in pediatric recipients, particularly in children under five years of age. A Pediatric Nephrology Research Consortium (PNRC) study found a 32% rate of MMF modifications (discontinuation or conversion to another medication) [25]. In the present study, a similar rate (27.3%) was observed. The rate was even higher in children aged < 2 years (75.0%) and 2–5 years (55.6%). Modifications were mainly antimetabolite discontinuations. In pediatric recipients, the reasons for MMF modifications are gastrointestinal symptoms, leucopenia, and transplant-associated viremias [17]. Young recipients (aged < 6 years) are shown to have numerically, though not statistically significantly, more adverse effects [26], which explains the more frequent need for MMF modifications. The definition of drug modification in the present study did not include dose reduction. If dose reductions are added, the modifications are observed in almost all recipients under five years of age [17].

The frequent need for MMF modifications is challenging and raises a concern of graft well-being. In NAPRTCS data, MMF was associated with a higher risk for late rejections in pediatric recipients [27]. The impact of MMF modification on rejection risk was examined in two retrospective pediatric studies [17, 25]. The Danish single-center study found no increase in the number of rejections following MMF modifications (including dose reduction), whereas the PNRC study reported more antibody-mediated rejections in MMF-modified recipients. The number of acute cellular rejections did not differ in the PNRC study either [25]. In the present study, the age-adjusted risk of rejection did not differ between the MMF-modified and the MMF-continued groups; however, a trend toward a lower risk of rejection was noticed in the MMF-modified group, possibly indicating a higher threshold for MMF modifications in patients who experienced an early rejection. The risk of rejection is highest within the first year after KT; MMF modifications did not increase late rejection risk beyond that level. No further conclusions regarding rejection risk can be drawn from the present study due to the small size of the MMF-modified group, the annual nature of data reporting, and the lack of detailed rejection classification.

To the best of our knowledge, this is the first pediatric study presenting graft survival data after MMF modifications. A large adult study found decreased graft survival at 4-year follow-up only in recipients who discontinued MMF due to gastrointestinal symptoms (70.2%) compared to recipients who continued with MMF (87.1%) [28]. In contrast, our data showed comparable graft survival for the MMF-modified and the MMF-continued groups between 2 and 7.5 years post-KT. The favorable outcomes observed in our cohort may indicate a lower need for immunosuppression in young recipients. In addition, the differences in sample size and the absence of stratification by reason for MMF modification may have obscured potential differences in outcomes.

Infant recipients are in many ways a special KT group. The Nordic KT population is rather exceptional with a large number of young recipients suffering from congenital nephrotic syndrome [3]. Young KT recipients are more likely to receive CsA as a primary CNI and experience modification of MMF treatment. Importantly, a previous analysis of NPRTSG data showed better long-term graft survival in infant recipients compared with older children and no significant association between the type of CNI and graft survival [3]. The present study reveals that most CsA-treated children are converted to Tac rather early. Still, with the current practice including CsA to Tac conversion, the outcome in infant recipients is excellent as published previously [3]. In addition, a recent registry study revealed that young recipients have fewer rejections compared with adolescents [29]. Moreover, deciding on IS for very young recipients cannot be based only on effectiveness; infections, adverse effects, and malignancies should be considered as well. Young children suffer from more infections after transplantation, also severe infections, compared to school-aged or adolescent recipients [29]. Further, the recipient’s young age as well as the recipient’s Epstein-Barr virus (EBV) seronegativity combined with the donor’s EBV seropositivity are well-defined risk factors for PTLD [16, 20]. Infant recipients often face all these PTLD risk factors. In children, PTLD risk is biphasic but highest within the first year after transplantation [30–32]. Considering infant recipients’ higher infection and PTLD risk as well as their lower rejection profile and previous excellent outcomes, lower total IS within the first years after transplantation might be beneficial for this special group of recipients.

The present study has some limitations. This is a retrospective, registry-based study, and the follow-up data are not complete. Due to the annual nature of maintenance IS data collection, temporary drug changes could not be analyzed and were likely not detected in most cases. Reasons for changes in IS are not included in the follow-up data. Concurrent rejection and PTLD were the only causes for changing immunosuppression that could be identified from the registry. However, we requested and received additional data for the subgroup of CsA-to-Tac converted patients. In addition, the protocol for maintenance immunosuppression differed between centers. Although our study does not include data from the few most recent years, we believe the findings remain relevant as there have been no major changes in treatment protocols during that time. Furthermore*,* EBV viremias and serostatus were reported only in a limited number of patients, and the number of PTLD cases did not allow adjustment for confounders.

In conclusion, our registry study shows that modifications in the maintenance IS are common among pediatric KT recipients, particularly CsA conversion to Tac and young children’s MMF modifications. This merits consideration in clinical practice. When initiating IS with MMF for a very young recipient or with CsA for a recipient of any age, the clinician should be aware that treatment modifications are often necessary. These medications need not be avoided but rather utilized with preparedness for adjustments based on individual patient needs.

## Supplementary Information

Below is the link to the electronic supplementary material.

Graphical abstract (PPT 174 KB)

ESM 2 (PDF 98.1 KB)

## Data Availability

Each member hospital within Scandiatransplant remains the owner of its data; therefore, Scandiatransplant data cannot be shared with any third party.

## Abbreviations

ATG: Antithymocyte globulin
Aza: Azathioprine
CAKUT: Congenital anomalies of the kidney and urinary tract
CNI: Calcineurin inhibitor
CsA: Cyclosporine A
EBV: Epstein–Barr virus
GFR: Glomerular filtration rate
HLA: Human leukocyte antigen
IFTA: Interstitial fibrosis and tubular atrophy
IQR: Interquartile range
IS: Immunosuppression
KT: Kidney transplantation
mGFR: Measured glomerular filtration rate
MMF: Mycophenolate mofetil
mTOR: Mammalian target of rapamycin
NPRTSG: Nordic Pediatric Renal Transplant Study Group
PNRC: Pediatric Nephrology Research Consortium
PTLD: Post-transplant lymphoproliferative disorder
SD: Standard deviation
Tac: Tacrolimus
