# Supplementary material for: Changes in maintenance immunosuppression after pediatric kidney transplantation—a report from the Nordic pediatric kidney transplantation registry
Source: Pediatr Nephrol. 2025 Nov 13;41(2):547–56. doi: 10.1007/s00467-025-07030-7 (PMC12727704; doi:10.1007/s00467-025-07030-7)
Supplement: Supplementary file 1 — Graphical abstract (PPT 174 KB) [file 467_2025_7030_MOESM1_ESM.ppt]

## Slide 1
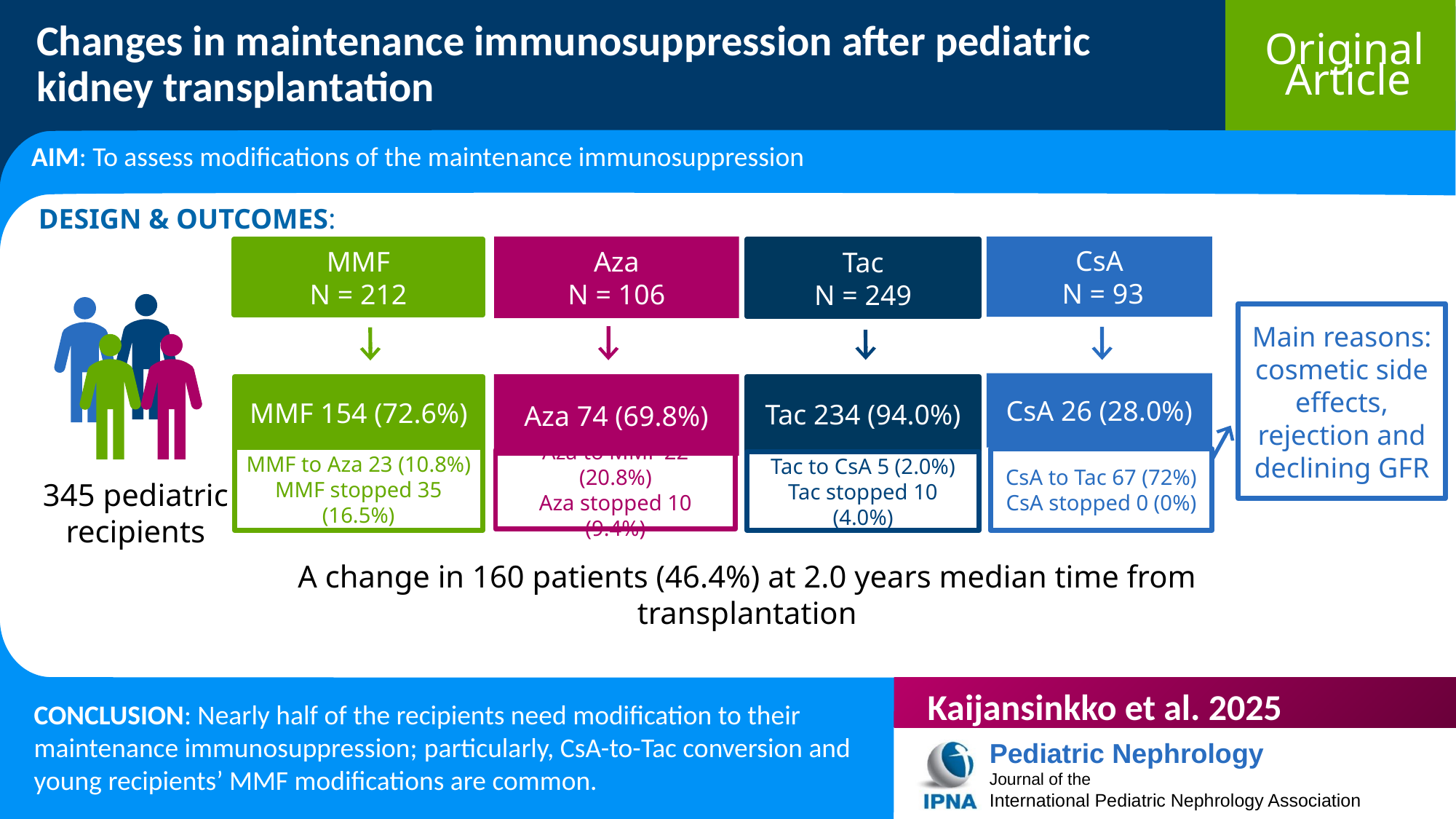

Changes in maintenance immunosuppression after pediatric kidney transplantation
AIM: To assess modifications of the maintenance immunosuppression
DESIGN & OUTCOMES:
Aza
N = 106
CsA
 N = 93
MMF
N = 212
Tac
N = 249
Main reasons: cosmetic side effects, rejection and declining GFR
CsA 26 (28.0%)
Aza 74 (69.8%)
MMF 154 (72.6%)
Tac 234 (94.0%)
MMF to Aza 23 (10.8%)
MMF stopped 35 (16.5%)
CsA to Tac 67 (72%)
CsA stopped 0 (0%)
Aza to MMF 22 (20.8%)
Aza stopped 10 (9.4%)
Tac to CsA 5 (2.0%)
Tac stopped 10 (4.0%)
345 pediatric recipients
A change in 160 patients (46.4%) at 2.0 years median time from transplantation
Kaijansinkko et al. 2025
CONCLUSION: Nearly half of the recipients need modification to their maintenance immunosuppression; particularly, CsA-to-Tac conversion and young recipients’ MMF modifications are common.
