# Supplementary material for: Changes in maintenance immunosuppression after pediatric kidney transplantation—a report from the Nordic pediatric kidney transplantation registry
Source: Pediatr Nephrol. 2025 Nov 13;41(2):547–56. doi: 10.1007/s00467-025-07030-7 (PMC12727704; doi:10.1007/s00467-025-07030-7)
Supplement: Supplementary file 2 — (PDF 98.1 KB) [file 467_2025_7030_MOESM2_ESM.pdf]

Supplementary information

Pediatric Nephrology

Changes in maintenance immunosuppression after pediatric kidney transplantation – A report from the Nordic pediatric kidney transplantation registry

Henna Kaijansinkko[1,2], Juuso Tainio, Anna Bjerre, Ann Christin Gjerstad, Ilse D. S. Weinreich, Hannu Jalanko, Lars Wennberg, Susanne Westphal Ladfors, Helle Charlotte Thiesson, Zivile Bekassy, Søren Schwartz Sørensen, Timo Jahnukainen

[1]Tampere University Hospital, Department of Pediatrics, Tampere, Finland.

[2]Department of Pediatric Nephrology and Transplantation, New Children's Hospital, University of Helsinki and Helsinki University Hospital, Helsinki, Finland.

Corresponding Author: Henna Kaijansinkko

Email: henna.kaijansinkko@gmail.com

Supplementary Table 1 Initial calcineurin inhibitors and antimetabolites according to sum mismatches.

	Sum mismatches						
	0 (n = 8)	1 (n = 33)	2 (n = 85)	3 (n = 116)	4 (n= 48)	5 (n = 19)	6 (n = 10)
CNI, n (%)							
CsA	2 (25.0)	4 (12.1)	27 (31.8)	39 (33.6)	8 (16.7)	2 (10.5)	1 (10.0)
Tac	6 (75.0)	29 (87.9)	56 (65.9)	77 (66.4)	39 (81.3)	17 (89.5)	9 (90.0)
None	0 (0.0)	0 (0.0)	2 (2.4)	0 (0.0)	1 (2.1)	0 (0.0)	0 (0.0)
Antimetabolite, n (%)							
Aza	2 (25.0)	8 (24.2)	27 (31.8)	33 (28.4)	12 (25.0)	3 (15.8)	3 (30.0)
MMF	5 (62.5)	22 (66.7)	53 (62.4)	70 (60.3)	33 (68.8)	16 (84.2)	5 (50.0)
None	1 (12.5)	3 (9.1)	5 (5.9)	13 (11.2)	3 (6.3)	0 (0.0)	2 (20.0)

CNI; calcineurin inhibitor, CsA; cyclosporine A, Tac; tacrolimus Aza; azathioprine, MMF; mycophenolate mofetil.

No mismatch data in 26 patients.

Supplementary Table 2 Number of patients according to the initial immunosuppression combination and the number of rejections

	Number of rejections *				
	0	1	2	3	4
IS combination, n (%)					
CsA + Aza	48 (67.6)	14 (19.7)	6 (8.5)	2 (2.8)	1 (1.4)
CsA + MMF	15 (88.2)	2 (11.8)	0 (0)	0 (0)	0 (0)
Tac + Aza	31 (91.2)	1 (2.9)	1 (2.9)	1 (2.9)	0 (0)
Tac + MMF	160 (84.2)	23 (12.1)	4 (2.1)	1 (0.5)	2 (1.1)

IS; immunosuppression, CsA; cyclosporine A, Aza; azathioprine, MMF; mycophenolate, Tac; tacrolimus

* Kruskal-Wallis test (P = 0.005). Significant differences in pairwise comparisons between groups CsA + Aza vs. Tac + MMF (P = 0.013) using the Mann-Whitney U-test with Bonferroni adjustment. No rejection data on 3 patients.

Supplementary Table 3 Initial maintenance calcineurin inhibitor and prevalence of post-transplant lymphoproliferative disorder in all KT recipients and subgroups divided by the age at transplantation

	< 2 years<br>CsA n = 42<br>Tac n = 29	2-5 years<br>CsA n = 14<br>Tac n = 44	5-16 years<br>CsA n = 37<br>Tac n = 149	Total<br>CsA n = 93<br>Tac n = 222
CsA: PTLD n (%)	2 (4.8)	0 (0.0)	0 (0.0)	2 (2.2)
Tac: PTLD n (%)	3 (10.3)	2 (4.5)	3 (2.0)	8 (3.6)

CsA; cyclosporine A, Tac; tacrolimus, PTLD; post-transplant lymphoproliferative disorder. Significantly less PTLD cases in the oldest patient group compared to the youngest group (5–16 years vs. < 2 years OR = 0.22; 95% CI 0.05–0.93; p = 0.040); no difference between the two youngest age groups (2–5 years vs. < 2 years OR = 0.48; 95% CI 0.09–2.56; P = 0.389). No difference in the number of PTLD cases according to the initial CNI (CsA vs. Tac OR 0.59; 95% CI 0.12–2.82; P = 0.507). No malignancy data in 27 patients.

Supplementary Table 4 Number of patients according to the steroid use and the number of rejections; the presented data includes only recipients with tacrolimus + mycophenolate immunosuppression

	Number of rejections *				
	0	1	2	3	4
Use of steroids, n (%)					
Early steroid withdrawal	34 (89.5)	3 (7.9)	1 (2.6)	0 (0)	0 (0)
Late steroid withdrawal	15 (75.0)	4 (20.0)	0 (0)	1 (5.0)	0 (0)
Long-term steroid treatment	109 (84.5)	15 (11.6)	3 (2.3)	0 (0)	2 (1.6)

Kruskal-Wallis test (P = 0.359). No rejection data in 1 patient.

Supplementary Table 5 Changes in initial maintenance immunosuppression; the data are presented in subgroups according to transplant era

Immunosuppressive drug	2005–2010<br>n = 195	2011–2016<br>n = 150	P-value
Calcineurin inhibitors			
Cyclosporine A, n (%)			p = 0.818[a]
No change	15 (29.4)	11 (26.2)	
CsA → Tac	36 (70.6%)	31 (73.8)	
CNI stopped	0 (0)	0 (0)	
Tacrolimus, n (%)			p = 1.000[a]
No change	135 (93.8)	99 (94.3)	
Tac → CsA	1 (0.7)	4 (3.8)	
CNI stopped	8 (5.6)	2 (1.9)	
No CNI, n (%)	0 (0)	3 (2.0)	
Antimetabolites			
MMF, n (%)			p = 0.442[a]
No change	72 (69.9)	82 (75.2)	
MMF → Aza	13 (12.6)	10 (9.2)	
Antimetabolite stopped	18 (17.5)	17 (15.6)	
Aza, n (%)			p = 0.514[a]
No change	45 (67.2)	29 (74.4)	
Aza → MMF	14 (20.9)	8 (20.5)	
Antimetabolite stopped	8 (11.9)	2 (5.1)	
No antimetabolite, n (%)	25 (12.8)	2 (1.3)	

CsA; cyclosporine A, CNI; calcineurin inhibitor, Tac; tacrolimus, MMF; mycophenolate mofetil, Aza; azathioprine

[a] Fisher's exact test. No statistically significant difference in changes (no change vs. change) between transplant eras.

Supplementary Table 6 Concomitant rejection in overall immunosuppression changes and with changes of single immunosuppressants.

	Concurrent rejection n (%)	No concurrent rejection n (%)
IS change (overall)	27 (17.0)	132 (83.0)
CNI change		
CsA → Tac n = 67	16 (23.9)	51 (76.1)
Tac → CsA n = 5	2 (40.0)	3 (60.0)
Antimetabolite change		
Aza → MMF n = 22	6 (27.3)	16 (72.7)
MMF → Aza n = 23	2 (8.7)	21 (91.3)

IS; immunosuppression, CNI; calcineurin inhibitor, CsA; cyclosporine A, Tac; tacrolimus, Aza; azathioprine, MMF; mycophenolate mofetil
